# Necrotizing Pneumonia Caused by Community-acquired Methicillin-resistant *Staphylococcus aureus* Following Influenza

**DOI:** 10.31662/jmaj.2025-0093

**Published:** 2025-05-30

**Authors:** Maiko Awashima, Yoshiko Kichikawa, Ko Suzuki, Hideyuki Horikoshi

**Affiliations:** 1 Department of Internal Medicine, Self-Defense Forces Central Hospital, Tokyo, Japan; 2 Department of Internal Medicine, Mishuku Hospital, Tokyo, Japan; 3 Department of Respiratory Medicine, Mishuku Hospital, Tokyo, Japan

**Keywords:** community-acquired methicillin-resistant *Staphylococcus aureus*, CA-MRSA, CA-MRSA pneumonia, necrotizing pneumonia

A 39-year-old female was presented to our hospital with fever, dyspnea, and chest pain. She had been diagnosed with influenza virus infection and had received antiviral drugs two days earlier. Despite this, she experienced difficulty maintaining adequate oxygenation on room air. She was administered oxygen using a reservoir mask at 15 L/min. Chest computed tomography (CT) revealed bilateral infiltrates (Figure 1), and methicillin-resistant *Staphylococcus aureus* (MRSA) was detected in her sputum. By the 10th day of hospitalization, chest CT showed multiple cavitary lesions (Figure 2). She was diagnosed with necrotizing pneumonia caused by community-acquired MRSA (CA-MRSA) ^(1)^. Following the administration of Linezolid, her clinical condition improved. In Japan, reports of CA-MRSA pneumonia are rare, but most of them involve severe pneumonia with the destruction of lung tissue. Unlike healthcare-associated MRSA pneumonia, CA-MRSA pneumonia has a tendency for cavitation within infiltrates ^(2)^. Therefore, it is necessary to include CA-MRSA pneumonia in the differential diagnosis when encountering pneumonia with multiple cavitary lesions.

**Figure 1.**
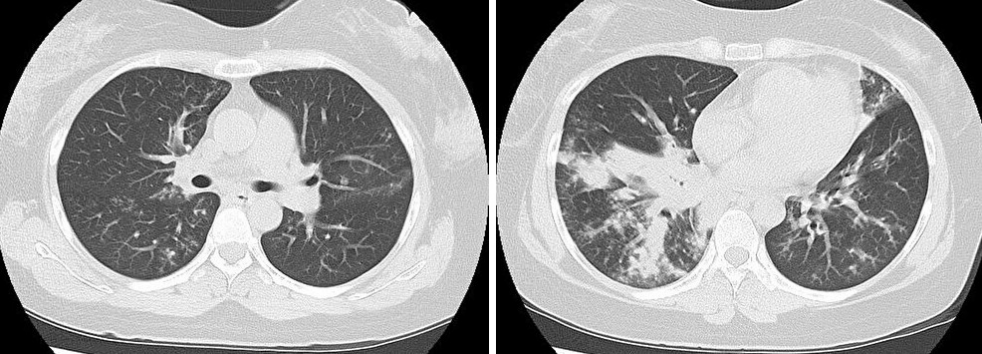
Chest computed tomography performed at the time of admission, showing infiltrates in both lungs.

**Figure 2.**
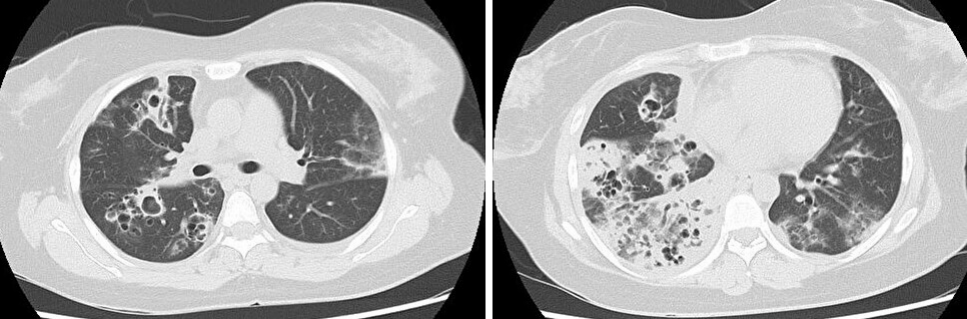
Chest computed tomography obtained on the 10th day of hospitalization, revealing multiple cavitary lesions.

## Article Information

### Conflicts of Interest

None

### Author Contributions

Maiko Awashima wrote the manuscript, and the other authors revised it.

### Approval by Institutional Review Board (IRB)

This study did not require IRB approval.

### Informed Consent

Consent was obtained from the patient.
